# Characteristics and outcome of COVID-19 patients admitted to the ICU: a nationwide cohort study on the comparison between the consecutive stages of the COVID-19 pandemic in the Netherlands, an update

**DOI:** 10.1186/s13613-023-01238-2

**Published:** 2024-01-16

**Authors:** Fabian Termorshuizen, Dave A. Dongelmans, Sylvia Brinkman, Ferishta Bakhshi-Raiez, M. Sesmu Arbous, Dylan W. de Lange, Bas C. T. van Bussel, Nicolette F. de Keizer, the Dutch COVID-19 Research Consortium, M.G.W. Barnas, D.P. Boer, R.J. Bosman, G.B. Brunnekreef, M. de Graaff, R.M. de Jong, A.R. de Meijer, W. de Ruijter, R. de Waal, A. Dijkhuizen, T.P.J. Dormans, A. Draisma, I. Drogt, B.J.W. Eikemans, P.W.G. Elbers, J.L. Epker, M.L. Erkamp, B. Festen-Spanjer, T. Frenzel, L. Georgieva, N.C. Gritters, I.Z. Hené, S.H.A. Hendriks, M. Hoeksema, J.W.M. Holtkamp, M.E. Hoogendoorn, C.J.G.M. Jacobs, I.T.A. Janssen, H. Kieft, M.P. Koetsier, T.J.J. Koning, H. Kreeftenberg, N. Kusadasi, J.A. Lens, J.G. Lutisan, D.J. Mehagnoul-Schipper, D. Moolenaar, F. Nooteboom, R.V. Pruijsten, D. Ramnarain, A.C. Reidinga, E. Rengers, A.A. Rijkeboer, T. Rijpstra, F.W. Rozendaal, R.M. Schnabel, V.M. Silderhuis, J.J. Spijkstra, P.E. Spronk, L.C. Urlings-Strop, A.E. van den Berg, R. van den Berg, I.C.C. van der Horst, P.H.J. Van der Voort, E.M. van Driel, L. van Gulik, F.M. van Iersel, M. van Lieshout, J.A.H. van Oers, E.R. van Slobbe-Bijlsma, M. van Tellingen, D.P. Verbiest, D.J. Versluis, E. Verweij, M. de Vrolijk-Mos, R.M.J. Wesselink

**Affiliations:** 1 National Intensive Care Evaluation (NICE) Foundation, Postbus 23640, 1100 EC Amsterdam, The Netherlands; 2 Amsterdam UMC, Department of Medical Informatics, University of Amsterdam, Amsterdam Public Health Research Institute, Meibergdreef 9, 1105 AZ Amsterdam, The Netherlands; 3 Amsterdam UMC, Department of Intensive Care Medicine, University of Amsterdam, Meibergdreef 9, 1105 AZ Amsterdam, The Netherlands; 4 Department of Intensive Care Medicine, Leiden University Medical Center, Albinusdreef 2, 2333 ZA Leiden, The Netherlands; 5 University Medical Center, Department of Intensive Care Medicine, University of Utrecht, Heidelberglaan 100, 3584 CX Utrecht, The Netherlands; 6 Department of Intensive Care Medicine, Maastricht University Medical Centre +, P. Debyelaan 25, 6229 HX Maastricht, The Netherlands; 7 Maastricht University, Care and Public Health Research Institute (CAPHRI), Cardiovascular Research Institute (CARIM), Universiteitssingel 40, 6229 ER Maastricht, The Netherlands

**Keywords:** COVID-19, Coronavirus, Mortality, Outcome, Intensive Care, Critical Care

## Abstract

**Background:**

Previously, we reported a decreased mortality rate among patients with COVID-19 who were admitted at the ICU during the final upsurge of the second wave (February–June 2021) in the Netherlands. We examined whether this decrease persisted during the third wave and the phases with decreasing incidence of COVID-19 thereafter and brought up to date the information on patient characteristics.

**Methods:**

Data from the National Intensive Care Evaluation (NICE)-registry of all COVID-19 patients admitted to an ICU in the Netherlands were used. Patient characteristics and rates of in-hospital mortality (the primary outcome) during the consecutive periods after the first wave (periods 2–9, May 25, 2020–January 31, 2023) were compared with those during the first wave (period 1, February–May 24, 2020).

**Results:**

After adjustment for patient characteristics and ICU occupancy rate, the mortality risk during the initial upsurge of the third wave (period 6, October 5, 2021–January, 31, 2022) was similar to that of the first wave (OR_adj_ = 1.01, 95%-CI [0.88–1.16]). The mortality rates thereafter decreased again (e.g., period 9, October 5, 2022–January, 31, 2023: OR_adj_ = 0.52, 95%-CI [0.41–0.66]). Among the SARS-CoV-2 positive patients, there was a huge drop in the proportion of patients with COVID-19 as main reason for ICU admission: from 88.2% during the initial upsurge of the third wave to 51.7%, 37.3%, and 41.9% for the periods thereafter. Restricting the analysis to these patients did not modify the results on mortality.

**Conclusions:**

The results show variation in mortality rates among critically ill COVID-19 patients across the calendar time periods that is not explained by differences in case-mix and ICU occupancy rates or by varying proportions of patients with COVID-19 as main reason for ICU admission.

The consistent increase in mortality during the initial, rising phase of each separate wave might be caused by the increased virulence of the contemporary virus strain and lacking immunity to the new strain, besides unmeasured patient-, treatment- and healthcare system characteristics.

**Supplementary Information:**

The online version contains supplementary material available at 10.1186/s13613-023-01238-2.

## Introduction

In the early phases of the COVID-19 pandemic, healthcare systems worldwide were confronted with large numbers of critically ill patients with an—at that time—unknown respiratory infection. During the subsequent epidemic upsurges, various reports on the characteristics of patients and treatment outcomes were published, often with preliminary conclusions [1–6].

Previously, we reported on the characteristics and outcomes of COVID-19 patients admitted to the ICU in the Netherlands [7–9]. In accordance with other studies [10, 11], high mortality rates among critically ill patients with COVID-19 compared to the rates for other critically ill patients with viral pneumonia were found [7, 9]. Fortunately, the data showed a decreased in-hospital mortality rate during the final upsurge of the second wave in the Netherlands (February–June 2021) [8]. This was interpreted as a possible effect of increasingly appropriate treatments, effective logistic and organizational measures that were taken and more efficient care. If so, we may expect that this decrease will have continued or mortality rates will have plateaued during the upsurges of COVID-19 following the second wave. Alternatively, differences in mortality rate may be associated with seasonal influences, as was recently found in a cohort of cardiac surgery patients admitted at the ICU [12].

In the present study, we updated our previous report, including patients up to January 2023.

We examined whether the decrease in mortality rate among ICU patients infected with SARS-CoV-2 persisted during the third wave (October, 2021–May, 2022) and the phases with decreasing incidence of COVID-19 thereafter (the period in-between: May–October, 2022, and the ‘endemic phase’: October, 2022–January, 2023) and brought up to date the information on patient- and treatment characteristics.

The aim was to evaluate whether there is evidence for an improvement in the quality of care for COVID-19 patients at the ICU, as indicated by a lower in-hospital mortality (primary outcome), lower relocation rates, and a shorter duration of ICU stay (secondary outcomes) in the course of the pandemic waves, the periods in—between and the endemic phase thereafter.

## Methods

### Data

Details on the data used are described in our previous study [8]. In summary, the National Intensive Care Evaluation (NICE) registry is a quality registry in which all Dutch ICUs participate [13]. This registry includes prospectively collected demographic and clinical data of all patients admitted to an ICU extracted from the ICU’s electronic health record (EHR). The purpose of NICE is to provide feedback on performance indicators to ICUs, thus enabling ICUs to monitor and improve their quality of care. From the start of the COVID-19 outbreak in the Netherlands, the Dutch government requested all ICUs to record all suspected and confirmed COVID-19 patients admitted to the ICU. Therefore, the existing NICE data infrastructure was expanded with a module allowing daily recording of admission- and discharge dates, and survival status at ICU- and hospital discharge of COVID-19 patients that could be linked to the clinical data once these were uploaded from the EHRs. NICE data infrastructure allowed that COVID-19 patients could be accurately tracked throughout subsequent hospital admissions. This made it possible to take transfers to other hospitals during an ongoing treatment episode into account, thus, enabling the evaluation of the complete treatment trajectory of an individual COVID-19 patient.

The Medical Ethics Review Committee of the Academic Medical Center waived the need for informed consent [reference number W21_091 # 21.102].

A confirmed COVID-19 patient was defined as follows: a positive SARS-CoV-2 Reverse transcription polymerase chain reaction (RT-PCR) on a nasopharyngeal swab or a CT-scan consistent with COVID-19 (i.e., a CO-RADS score of ≥ 4 in combination with the clinical diagnosis viral pneumonia) [14]. Few patients were diagnosed with COVID-19 on the basis of a CT-scan. During the first wave and the first period in-between, the number of patients diagnosed with CT-scan was 56 (2%) and 11 (2.2%). Thereafter, this percentage declined to around 0.2–0.5% (data not shown).

**Fig. 1:**
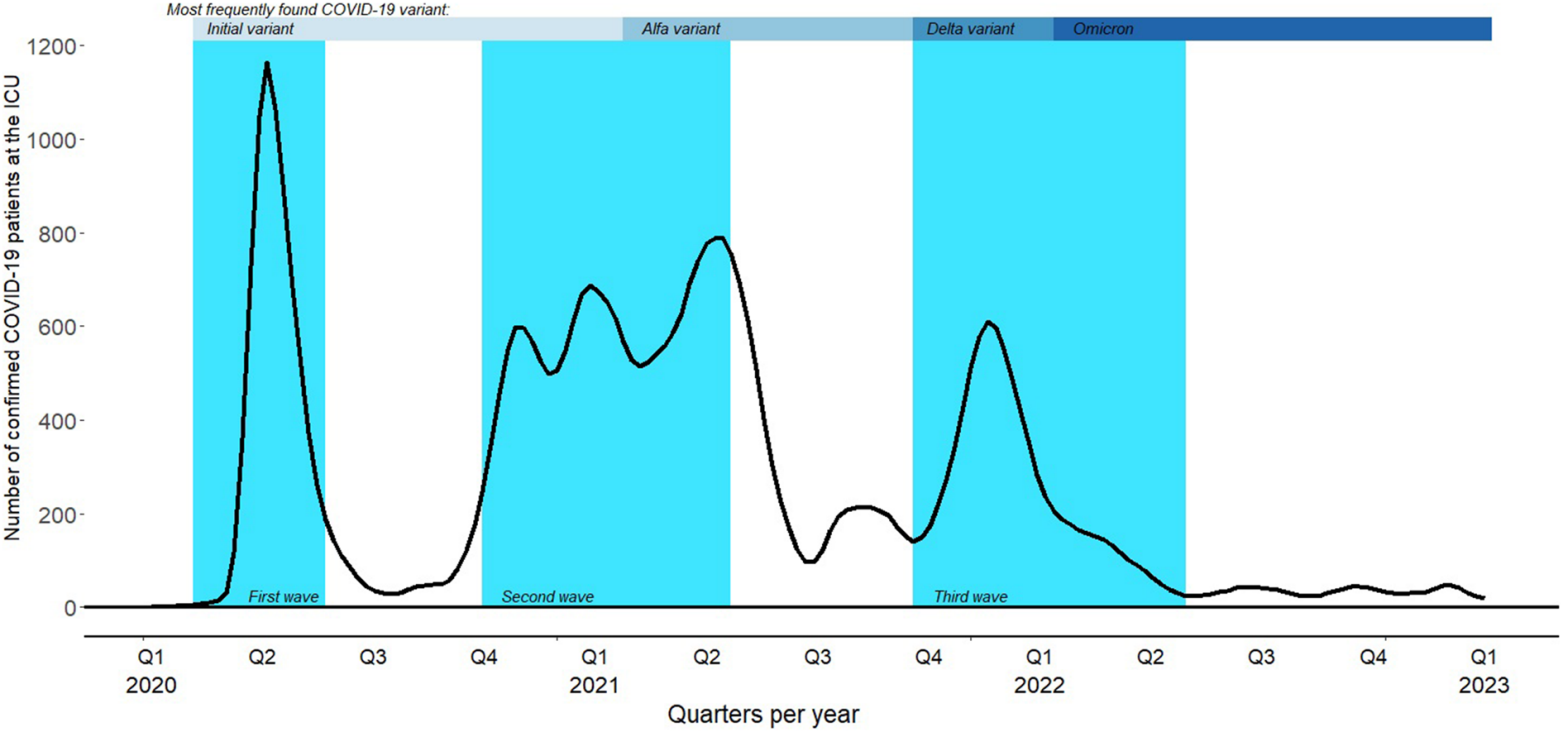
Number of COVID-19 patients present at the ICU during the pandemic waves in the Netherlands

In 2020, 2021, and 2022, three major waves of ICU admissions due to SARS-CoV-2 infections were observed in the Netherlands with distinct periods in between (Fig. 1 and Additional file 1: Figure S1). Calendar time (which is the exposure variable in the present analysis) was categorized into the following time periods:

1. The first wave (February 1-May 24, 2020, initial variant predominates),
2. The period between the waves (May 25 – October 4, 2020),
3. The second wave—first and second upsurge (October 5, 2020–January 31, 2021),
4. The second wave—final upsurge (February 1, 2021–May 24, 2021, alfa variant),
5. The period between the waves (May 25, 2021–October 4, 2021),
6. The third wave–initial upsurge (October 5, 2021–January 31, 2022, delta variant),
7. The third wave—second part (February 1–May 24, 2022, omicron variant),
8. The period after the third wave (May 25, 2022–October 4, 2022), and
9. The period of absence of a fourth wave, which is called ‘the endemic phase’ (October 5, 2022–January 31, 2023).

Categorization was done on the basis of visual inspection and with the aim to define periods that were consistently defined in terms of identical months and days across the subsequent calendar years. This was done to detect possible seasonal influences.

### Statistical analyses

The number of patients, patient- and treatment characteristics, duration of total ICU- and hospital stay (combining subsequent hospital and ICU stays within a treatment trajectory of an unique patient), occupancy rate, and relocation rate were described for the nine time periods (time periods mentioned above 1–9) separately. The ICU occupancy rate was calculated as the number of occupied beds at the day of ICU admission of the patient at the (first) ICU of admission as proportion of the daily average in 2019 specific for that ICU. The in-hospital mortality (primary outcome), that is death at the ICU or death at the hospital ward after ICU discharge, was estimated as percentage and analyzed in a multivariable logistic regression model with time period as the main independent variable. The Odds ratios (ORs) of in-hospital mortality (the primary outcome) during time periods 2–9 compared to time period 1 were adjusted for age, sex, Body Mass Index (BMI), the Acute Physiology and Chronic Health Evaluation (APACHE) IV mortality risk [15], and the ICU occupancy rate (the covariates) at the day of ICU admission. Patients with missing data on age (*N* = 1), BMI (*N* = 466), APACHE-IV probability (*N* = 84), occupancy rate (*N* = 89) were excluded from the logistic regression (*N* = 576, 3.1%). For those included (*N* = 18,196) the logistic regression was estimated both with inclusion and with exclusion of those patients who were still hospitalized at the time of database closure (July 25, 2023 (*N* = 214). Results were very similar. The length of ICU stay (the secondary outcome) was analyzed in a multivariable Cox regression analysis with ICU discharge as outcome event and death at the ICU as censoring event, and with adjustment for the same covariates. To take the possible influence of clustering of patients admitted at the same hospital on the results into account, both the logistic and the Cox regression models were re-run with an random intercept for hospital of admission. In case of transfer, the first hospital with a stay of at least 24 h was regarded as the hospital of admission for the estimation of the random intercept. As very similar results were found, the models without this random intercept are presented in the Results section. All analyses were performed using the R statistical environment (version 4.0.3) (R Foundation for Statistical Computing, Vienna, Austria). We report P-values and effect estimates with 95% confidence intervals and considered P < 0.05 statistically significant. The STROBE guidelines (https://www.equator-network.org/reporting-guidelines/strobe/) were followed to write the manuscript and report the results.

## Results

### Patient and treatment characteristics

From February 2020 until February 2023 there were 19,517 COVID-19 patients admitted to 78 Dutch ICUs. Of these, we excluded 745 patients since their clinical data were not (yet) available, leaving 18,772 patients (96.2%) for the final analyses.

The mean age decreased during period 2, that is the period between the first and second wave (from 63.2 to 61.9 years) and during period 5, that is, between the second and third wave (from 61.5 to 55.7 years), and increased again during the initial upsurge (period 6, 60.4 years) and second part (period 7, 62.6 years) of the third wave. The mean age increased further during the two time periods (period 8–9) thereafter (to 63.3 and 66.4 years) (Table 1).

**Table 1:** Baseline characteristics in group of patients in the COVID-19 registry with MDS record linkage (N = 18,772)

	1.Wave 1 01/02/2020- 24/05/2020	2.In-between 25/05/2020–04/10/2020	3.Wave 2.1 05/10/2020- 31/01/2021	4.Wave 2.2 01/02/2021- 24/05/2021	5.In-between 25/05/2021- 04/10/2021	6.Wave 3.1 05/10/2021- 31/01/2022	7.Wave 3.2 01/02/2022- 24/05/2022	8.In-between 25/05/2022- 04/10/2022	9.Endemic 05/10/2022- 31/01/2023
Number (N) of patients	2816	507	4470	4663	1292	3234	1239	606	690
with MDS linkage: N (%)	2740 (97.3)	490 (96.6)	4300 (96.2)	4602 (98.7)	1272 (98.5)	3074 (95.1)	1141 (92.1)	555 (91.6)	598 (86.7)
Mean age (years) (SD)	63.2 (11.3)	61.9 (12.8)	64.3 (11.3)	61.5 (11.7)	55.7 (14.4)	60.4 (13.2)	62.6 (14.7)	63.3 (15.9)	66.4 (13.8)
Gender: Male N (%)	1976 (72.1)	334 (68.2)	3068 (71.3)	3082 (67.0)	777 (61.1)	2049 (66.7)	688 (60.3)	338 (60.9)	389 (65.1)
Comorbidity N (%):									
Immune insufficiency	208 (7.6)	40 (8.2)	459 (10.7)	416 (9.0)	133 (10.5)	325 (10.6)	254 (22.3)	125 (22.5)	119 (19.9)
Renal insufficiency	73 (2.7)	19 (3.9)	252 (5.9)	153 (3.3)	44 (3.5)	137 (4.5)	99 (8.7)	48 (8.6)	66 (11)
Respiratory insufficiency	306 (11.2)	50 (10.2)	633 (14.7)	561 (12.2)	129 (10.1)	331 (10.8)	209 (18.3)	106 (19.1)	147 (24.6)
Cardiovascular	34 (1.2)	7 (1.4)	84 (2.0)	68 (1.5)	20 (1.6)	37 (1.2)	42 (3.7)	21 (3.8)	26 (4.3)
Malignancy	66 (2.4)	13 (2.7)	144 (3.3)	88 (1.9)	31 (2.4)	80 (2.6)	104 (9.1)	56 (10.1)	55 (9.2)
Liver cirrhosis	3 (0.1)	2 (0.4)	22 (0.5)	23 (0.5)	8 (0.6)	20 (0.7)	13 (1.1)	8 (1.4)	7 (1.2)
Diabetes mellitus	522 (19.1)	146 (29.8)	1116 (26)	972 (21.1)	268 (21.1)	631 (20.5)	235 (20.6)	104 (18.7)	129 (21.6)
At least 1 of these N (%)	977 (35.7)	218 (44.5)	1998 (46.5)	1801 (39.1)	480 (37.8)	1155 (37.6)	618 (54.1)	297 (53.5)	352 (58.9)
Mean BMI (kg/m^2^) (SD)	28.7 (4.99)	29.39 (5.59)	29.42 (5.38)	30.05 (5.81)	30.31 (6.42)	29.36 (5.78)	27.66 (6.26)	26.4 (5.55)	27.12 (6.28)
BMI > 30 N (%)	843 (30.8)	180 (36.7)	1651 (38.4)	1974 (42.9)	558 (43.9)	1222 (39.8)	307 (26.9)	105 (18.9)	135 (22.6)
APACHE-IV 1st diagnosis:									
Viral pneumonia/ ARDS/	2550 (93.0)	424 (86.5)	3940 (91.7)	4337 (94.2)	1179 (92.7)	2710 (88.2)	589 (51.7)	207 (37.3)	251 (41.9)
Sars-Cov-2 N (%)									
Mean APACHE-III (SD)	60.21 (21.54)	60.14 (23.37)	63.74 (21.36)	60.09 (19.82)	58.16 (20.45)	61.52 (22.75)	66.27 (27.09)	70.82 (30.55)	69.98 (28.47)
1.ARDS (APACHE-IV) N(%)	436 (15.9)	56 (11.4)	445 (10.3)	617 (13.4)	159 (12.5)	425 (13.8)	58 (5.1)	8 (1.4)	15 (2.5)
2.PaO2/FiO2 ratio < 300	2379 (86.8)	394 (80.4)	3795 (88.3)	4202 (91.3)	1141 (89.7)	2697 (87.7)	787 (69.0)	351 (63.2)	386 (64.5)
1. and/ or 2. N (%)	2439 (89.0)	403 (82.2)	3809 (88.6)	4224 (91.8)	1146 (90.1)	2707 (88.1)	788 (69.1)	352 (63.4)	387 (64.7)

The percentage of patients without comorbidity at the time of ICU admission fluctuated between 64.3% (period 1, first wave) and 53.5% (period 3) up to and including the initial upsurge of the third wave (period 6: 62.4%). After period 6, this percentage decreased substantially to 45.8%, 46.5%, and 41.1% during the periods 7–9. During the periods 7–9, the prevalence of patients with comorbid immune insufficiency was substantially higher compared to the preceding periods, approximately 20% vs 10% and lower. A simultaneous and substantial increase in prevalence of other comorbid conditions was also found, especially of renal and respiratory insufficiency. For the definition of the various comorbidities shown in the Table, see Supplement Methods. The APACHE-III score (in which the severity of physiological disturbance, comorbidity, and age are taken into account, ranging from 0 to 299) fluctuated around 60.0 points up to and including the initial upsurge of the third wave (period 6), and, in accordance with the increasing mean age and prevalence of comorbid conditions, increased from the second part of the third wave (period 7) onwards to 70.8 during period 8, and 69.9 during period 9 (Table 1).

The percentage of patients with an APACHE-IV main reason for admission indicative of COVID-19 (that is viral pneumonia, ARDS, or Sars-Cov-2 infection) decreased considerably from values in the range 86.5–93.0% up to and including the initial upsurge of the third wave (period 6) to 51.7%, 37.3%, and 41.9% for the periods 7–9 thereafter (Table 1).

The percentages of patients treated with mechanical ventilation and/ or vasoactive drugs were high during the first wave (79.1% and 67.2%, resp.) and deceased substantially and remained lower for all periods thereafter (Table 2). The highest mean ICU occupancy rate was found during the first wave (183%). Lower values were found for the periods 2–7 thereafter. These values were larger than 100%, that is, higher than expected on the basis of the number of patients admitted in 2019 at the ICU concerned. During the last two periods from May, 2022 onwards (period 8 and 9), the mean occupancy rates were lower than 100%, that is, lower than expected. In accordance with these findings, the number of patients with at least one transfer to another hospital was high during the first wave (32.2%) and decreased during the periods thereafter to 5.7% in the last period (Table 2).

**Table 2:** Treatment characteristics and outcomes

	1.Wave 1 01/02/2020- 24/05/2020	2.In-between 25/05/2020–04/10/2020	3.Wave 2.1 05/10/2020- 31/01/2021	4.Wave 2.2 01/02/2021- 24/05/2021	5.In-between 25/05/2021- 04/10/2021	6.Wave 3.1 05/10/2021- 31/01/2022	7.Wave 3.2 01/02/2022- 24/05/2022	8.In-between 25/05/2022- 04/10/2022	9.Endemic 05/10/2022- 31/01/2023
N with MDS record linkage	2740	490	4300	4602	1272	3074	1141	555	598
Mean PaO2 (mmHg) t = 0 (SD)	84.97 (35.83)	78.6 (28.0)	76.7 (29.76)	76.82 (31.16)	76.33 (29.06)	77.27 (32.81)	87.55 (46.72)	89.68 (45.22)	86.07 (36.78)
Mech. ventil. t = 0 (N,%)	1320 (48.2)	100 (20.4)	1063 (24.7)	1118 (24.3)	278 (21.9)	831 (27.0)	358 (31.4)	202 (36.4)	211 (35.3)
Mech. ventil. t = 24 h (N,%)	2167 (79.1)	251 (51.2)	2503 (58.2)	2712 (58.9)	698 (54.9)	1791 (58.3)	566 (49.6)	266 (47.9)	282 (47.2)
Vasoactive drugs (N,%)	1842 (67.2)	213 (43.5)	2083 (48.4)	2159 (46.9)	532 (41.8)	1485 (48.3)	486 (42.6)	259 (46.7)	271 (45.3)
Acute renal failure (N,%)	252 (9.2)	34 (6.9)	289 (6.7)	278 (6.0)	47 (3.7)	214 (7.0)	140 (12.3)	78 (14.1)	85 (14.2)
Mean bed occupancy (SD)	1.82 (0.62)	1.08 (0.4)	1.51 (0.45)	1.65 (0.44)	1.07 (0.32)	1.34 (0.38)	1.01 (0.33)	0.86 (0.28)	0.99 (0.38)
Transfer to other hospital (N,%)	883 (32.2)	111 (22.7)	1206 (28)	1288 (28)	318 (25)	797 (25.9)	109 (9.6)	47 (8.5)	34 (5.7)
Mean length of pre ICU hospital stay in days (SD)	1.7 (2.9)	1.8 (4.3)	2.4 (10.3)	2.1 (3.2)	2.1 (3.4)	2.2 (7.8)	3.8 (28.9)	1.7 (5.6)	3.8 (35.5)
Mean length of stay in days ICU (SD)	20.6 (20.5)	16.0 (15.8)	17.2 (17.6)	16.3 (16.7)	15.8 (24.9)	15.4 (21.1)	9.4 (18.3)	7.6 (10.2)	7.0 (10.7)
Median length of stay ICU (IQR)	15 (8–28)	11 (5–12)	11 (6–23)	11 (6–21)	10 (5–20)	10 (5–20)	4 (2–11)	4 (2–9)	4 (2–8)
Mean length of stay in days hospital (SD)*^1^	41.6 (130.8)	41.8 (139.0)	36.1 (107.9)	36.0 (103.9)	28.5 (70.5)	27.2 (58.8)	19.9 (43.2)	18.3 (32.2)	16.0 (25.1)
Hospital death (N, %)	822 (30.0)	132 (26.9)	1392 (32.4)	1102 (23.9)	262 (20.6)	880 (28.6)	304 (26.6)	129 (23.2)	154 (25.8)

^*^^1^ calculated since data of admission at the ICU

### Outcome: in-hospital mortality

The comparatively low percentages of in-hospital death during the final upsurge of the second wave (period 4) (23.9%), as found in our previous study [8], and the period between the second and third wave (period 5) (20.6%) were followed by a considerably higher percentage during the initial upsurge of the third wave (period 6) (28.6%) (Table 2). During the periods 7–9 thereafter, the percentage of in-hospital mortality decreased again, ranging between 23.2 and 26.6%.

After adjustment for age, gender, BMI, APACHE-IV mortality risk, and occupancy rate, the odds of in-hospital death during the initial upsurge of wave 3 was very similar to the odds during wave 1 (OR_adj_ = 1.01, 95%-CI [0.89 -1.16]) and to the odds during the first upsurge of the second wave (OR_adj_ = 0.98, 95%-CI [0.87 -1.10]) (Table 3). During the second part of wave 3 (period 7), the in-hospital mortality again decreased (OR_adj_ = 0.70, 95%-CI [0.58–0.84]), comparable to the value found for the second upsurge of the second wave (OR_adj_ = 0.78, 95%-CI [0.69–0.88]). After wave 3, the adjusted in-hospital mortality further decreased to comparatively low values (OR_adj_ = 0.50 [0.39–0.65] and OR_adj_ = 0.52 [0.41–0.66] for periods 8 and 9, respectively. The parameter estimates related to all variables included in the multivariable model are shown in Additional file 1: Table S4. The parameter estimates for occupancy rate show no unfavorable effect of a high occupancy rate on the hospital mortality risk.

**Table 3:** Odds ratios of Hospital mortality during the consecutive stages of the COVID-19 pandemic (see Additional file 1: Table S4 and Additional file 1: Table S5 for details of the fully adjusted models and missing data)

	1.Wave 1 01/02/2020- 24/05/2020 Reference	2.In-between 25/05/2020–04/10/2020	3.Wave 2.1 05/10/2020- 31/01/2021	4.Wave 2.2 01/02/2021- 24/05/2021	5.In-between 25/05/2021- 04/10/2021	6.Wave 3.1 05/10/2021- 31/01/2022	7.Wave 3.2 01/02/2022- 24/05/2022	8.In-between 25/05/2022- 04/10/2022	9.Endemic 05/10/2022- 31/01/2023
Total (N = 18,772)									
1. Crude	1.00	0.83	1.12	0.74	0.60	0.95	0.86	0.73	0.83
	[1.00–1.00]	[0.67–1.04]	[1.00–1.24]	[0.66–0.82]	[0.51–0.70]	[0.84–1.06]	[0.74–1.01]	[0.58–0.90]	[0.68–1.02]
Wald X2, df, P-value								119.96, 8,	< 0.001
2. Adjusted for age, sex	1.00	0.91	0.99	0.78	0.83	1.04	0.75	0.54	0.55
BMI, and APACHE-IV risk	[1.00 – 1.00]	[0.71–1.17]	[0.88–1.11]	[0.70–0.88]	[0.70–1.00]	[0.91–1.18]	[0.63–0.89]	[0.43 – 0.69]	[0.44–0.70]
Wald X2, df, P-value								78.95, 8,	< 0.001
3. Adjusted for (…), and	1.00	0.86	0.98	0.78	0.79	1.01	0.70	0.50	0.52
ICU occupancy rate	[1.00 – 1.00]	[0.67–1.11]	[0.87–1.1]	[0.69–0.88]	[0.65–0.95]	[0.88–1.16]	[0.58–0.84]	[0.39–0.65]	[0.41–0.66]
Wald X2, df, P-value								81.97, 8,	< 0.001

Restricted to those with APACHE-IV diagnosis indicative of Covid-19 as main reason for ICU admission (N = 16,187)									
1. Crude	1.00	0.85	1.13	0.75	0.6	0.96	1.14	0.88	0.87
	[1.00 – 1.00]	[0.67–1.07]	[1.01–1.26]	[0.67–0.84]	[0.5–0.71]	[0.85–1.08]	[0.94–1.39]	[0.63–1.22]	[0.64–1.17]
Wald X2, df, P-value								112.62, 8,	< 0.001
2. Adjusted for age, sex	1.00	0.89	0.99	0.80	0.86	1.05	0.86	0.52	0.51
BMI, and APACHE-IV risk	[1.00 – 1.00]	[0.69–1.16]	[0.88–1.12]	[0.71–0.91]	[0.71–1.04]	[0.92–1.20]	[0.69–1.07]	[0.36–0.74]	[0.37–0.71]
Wald X2, df, P-value								51.838, 8,	< 0.001
3. Adjusted for (…), and	1.00	0.85	0.98	0.80	0.81	1.03	0.81	0.48	0.48
ICU occupancy rate	[1.00–1.00]	[0.65–1.11]	[0.87–1.11]	[0.71–0.91]	[0.67–0.99]	[0.89–1.18]	[0.64–1.01]	[0.33–0.69]	[0.34–0.68]
Wald X2, df, P-value								54.59, 8,	< 0.001

**Fig. 2:**
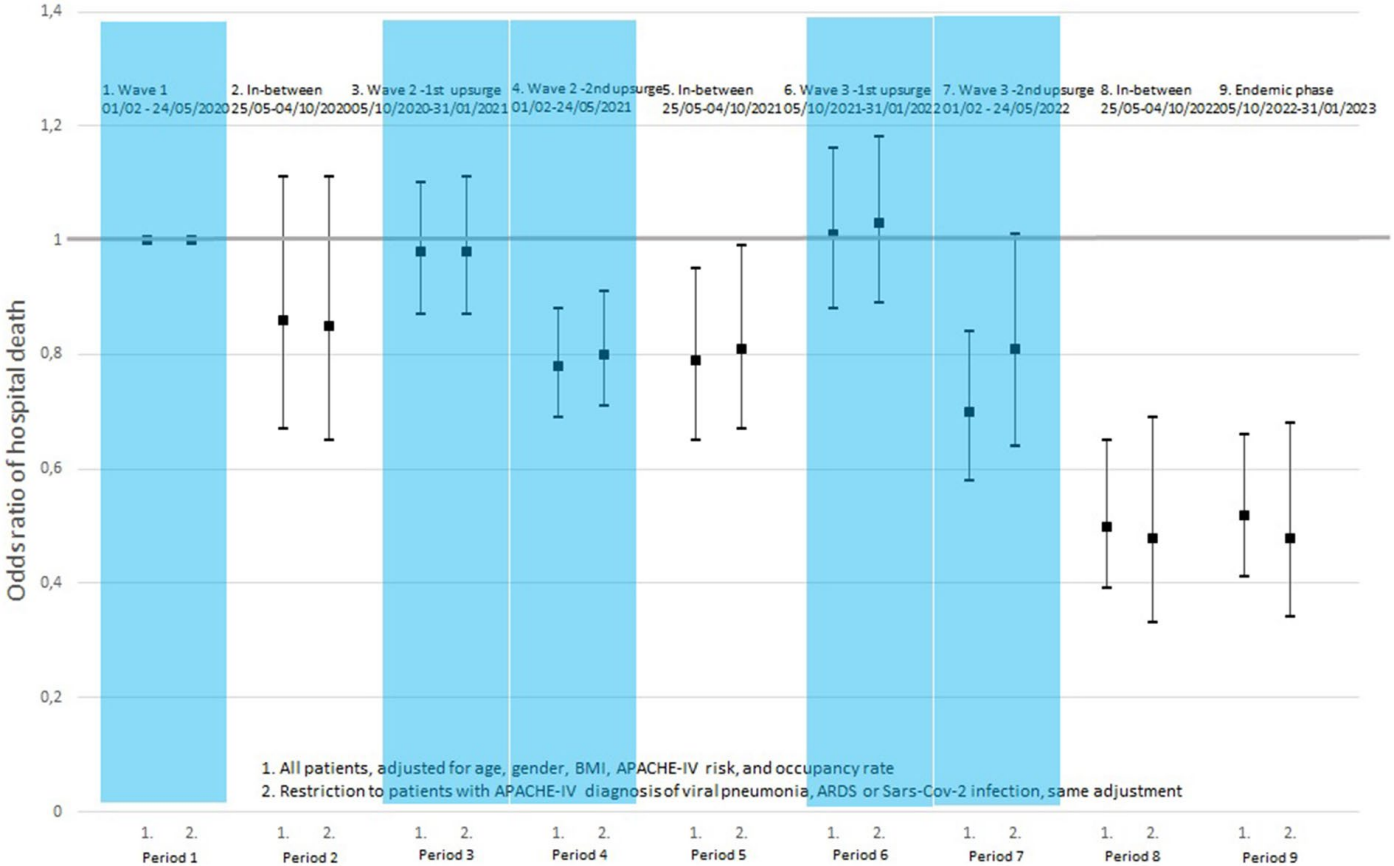
Odds ratios of Hospital mortality during the consecutive stages of the COVID-19 pandemic

Albeit the analysis was adjusted for a number of important clinical characteristics, the varying percentage of patients with an APACHE-IV main reason for admission indicative of COVID-19 was not taken into account. For this reason, the logistic regression analysis was repeated with a restriction to patients with an APACHE-IV main reason for admission indicative of COVID-19 (N = 16,187). The demographic, clinical, and treatment characteristics, and the crude in-hospital mortality rates related to this restricted group of COVID-19 patients are given in Additional file 1: Tables S1 and S2. Similar results of the logistic regression analysis on in-hospital mortality were found with again decreasing ORs from the second part of the third wave onwards (periods 7–9) (Table 3 and Additional file 1: Table S5). The adjusted ORs for the whole cohort and for the restricted group are shown for the separate time periods 1–9 in Fig. 2.

### Outcome: length of ICU stay

The mean length of stay at the ICU decreased considerably after the first wave from 20.6 days (period 1) to values in the range of 15.4–17.2 days for the periods 2–6. From the second part of the third wave onwards (period 7), the mean length of ICU stay further decreased to values in the range of 7.0–9.4 days (periods 7–9) (Table 2). This calendar time trend to lower values for ICU stay, and, thus, higher rates of ICU discharge was also found in a multivariable Cox regression analysis, both for the total group of patients (*N* = 18,772) and for the restricted group of patients with an APACHE-IV main reason for admission indicative of COVID-19 (*N* = 16,187) (Additional file 1: Table S3).

## Discussion

Our findings revealed that the adjusted mortality risk during the initial upsurge of the third wave (October 5, 2021–January 31, 2022) was similar to that of the first wave, that is, the mortality increased again following the considerably lower mortality rates found during the final upsurge of the second wave (February 1, 2021–May 24, 2021) [8]. After the initial upsurge of the third wave (from February, 2022 onwards), we observed again a decrease in the adjusted mortality rate, which was not followed by an increase in mortality rate during the period of low incidence in October, 2022–January, 2023, that is, the period in which a next (fourth) wave was anticipated but did not occur. There was a huge drop in the percentage of patients with an APACHE-IV main reason for admission indicative of COVID-19 after the initial upsurge of the third wave. We may assume that this drop was the reason for the decreasing and comparatively low mortality rates during the later periods. However, restricting the analysis to those patients in the COVID-19 registry with an APACHE-IV main reason for admission indicative of COVID-19 yielded similar results. Thus, we found consistently increased mortality rates during the rising phases of the consecutive epidemic COVID-19 waves, and consistently lower rates during the periods in-between and the evolving endemic phase thereafter.

### Interpretation of findings

The decreasing and low mortality rates (either with or without adjustment for age, gender, BMI, APACHE-IV mortality risk, and occupancy rate) during the later periods 7–9 are remarkable, as the mean age, the percentage of patients with comorbid conditions (such as immune deficiency), and the associated APACHE-IV mortality risk considerably increased. Thus, relatively less healthy patients were admitted from the second part of the third wave onwards. However, this did not result in a higher mortality rate. As the results were hardly influenced by adjustment for ICU occupancy rate, this finding was probably not the result of lower occupancy rates, which potentially led to higher quality of care during these later episodes. The absence of an increase in mortality rate during the period of low incidence in October, 2022–January 2023 (period 9) is in itself an important observation, as it suggests that the high mortality rates consistently found for the rising phases of the consecutive epidemic COVID-19 waves were not due to a predictable seasonal influence [12].

We also found a decrease in the mean length of stay at the ICU from above 20 days during the first wave to a range of 15–17 days during the next waves and periods in-between thereafter. We feel that this reflects the fact that the discharge of patients to the hospital wards became less challenging. As no data on individual therapeutic regimes were available we can only cautiously conclude that this was possibly associated with evolving treatment management strategies in ICUs as well as on wards and with the utilization of drugs such as tocilizumab [16, 17]. A further decrease in the mean length of stay for the last three periods 7–9 was observed. This may indicate that patients were less severely affected by the virus, in accordance with the decreasing mortality rates during these episodes. This decrease is important because it made it possible that with the same amount of beds more patients could be treated, thus relieving a part of the strain on the healthcare system.

The fluctuating mortality rates among critically ill COVID-19 patients across the calendar time periods, especially the re-emergence of the high mortality rate during the initial upsurge of the third wave, suggest that the previously found lower mortality rate for the final upsurge of the second wave [8] cannot be attributed without question to improved care and more effective logistic and organizational arrangements following the start of the pandemic. The increased mortality during the first upsurge of the third wave coincided with a much lower proportion of patients treated with mechanical ventilation and with vasoactive drugs, when compared to the first wave. This suggests that moving insights and changing treatment options did not prevent this increased mortality. As the higher mortality rates consistently coincide with the early, rising phases of the consecutive waves, and lower rates with the periods in between and the endemic phase thereafter we may assume an important role for the virulence of the COVID-19 strains itself. The introduction of the Omicron variant (which is known to have a much lower virulence) during the first months of 2022 onwards, the staying away of the fourth wave and the much lower mortality risk of Covid-19 patients admitted at the ICU from the early months of 2022 onwards support this notion [18]. This high virulence may imply preferential selection of those people who are more frail and vulnerable in the early phase of a wave, and, as a consequence, high mortality rates will ensue. In the subsequent phases, virulence will get weakened as to prevent eradication of the host population and, as a consequence, possibilities for transmission and survival of the virus. The importance for our findings is that the relevance of a higher or lower virulence obviously extends to those who are in urgent need of critical care and, thus, by definition are at high risk of death.

### Comparison with other studies

Our finding of an invariable high mortality among critically ill patients with COVID-19 is in accordance with a multicenter retrospective cohort study in Spain, Andorra, and Ireland [19]. In this study, the second/ third wave of July 2020–March, 2021 was compared with the first wave of February, 2020–June, 2020 with a break-down by month. No significant difference in adjusted ICU mortality rates between the two waves was found. Furthermore, there were higher mortality rates during the rising-up phase of the two subsequent waves, and the authors wondered whether this finding was caused by the rapid surge of patients, overload of the system, and impaired quality of care. In our analyses, we were able to adjust for ICU occupancy rate, and, as hardly any effect of this adjustment on the results was found, we may question this explanation, at least for our cohort. Furthermore, in a previous analysis of the NICE data we found that transferring critically ill COVID-19 patients in the Netherlands during peaks of high occupancy did not result in higher mortality rates during the first 180 days after ICU admission [20]. Our results are also in accordance with a nationwide register in Denmark [21]. No difference in adjusted 90-day mortality rates was found between patients admitted in the first wave (March–May, 2020) and those admitted thereafter (May 2020–June, 2021). This study is important, as the Danish healthcare system was not overwhelmed and the triage criteria were stable over the course of the pandemic. The invariable high in-hospital mortality was also found in a study in Australia, in which the three subsequent waves in the period February, 2020–November, 2021 were compared with each other [22]. The authors wondered whether the comparatively high in-hospital mortality during the third wave (June–November, 2021) may reflect increased overload of the ICU or the greater virulence of the Delta variant. An increase in 28-day mortality was found for the peak of the second wave after the lower mortality during the post-first-wave period among patients with COVID-19 in critical care in England [23]. This increase could be established after adjustment for both patient characteristics and occupancy levels. In a study among ICU patients in France, favorable effects of vaccination against COVID-19 on use of invasive mechanical ventilation and hospital death were found, but hospital death remained invariably high during the three surges between March 2020 and June 2021 [24]. The authors wondered whether this finding was due to the predominance of the high virulent alpha variant during the third surge and/ or the high strain at the ICUs. A number of smaller studies confirmed the invariably high mortality across the subsequent waves [2, 25]. A number of studies found favorable time trends to lower mortality rate among COVID-19 patients admitted at the ICU, but the data were restricted to the first wave [1, 3–5, 26, 27] or the differences in mortality between the waves were not adjusted for important patient characteristics [28, 29].

### Strengths and limitations

The main strength of our study was the presence of complete nationwide data of all critically ill COVID-19 patients admitted to the ICU during the subsequent waves of the pandemic and the endemic phase thereafter in the Netherlands, and the record linkage of these data to the NICE quality registry. We were able to combine multiple hospital stays of the same patient in case of hospital transfer(s) during an unique treatment trajectory. The calendar time axis was categorized in finely-meshed episodes to match both the epidemic upsurges followed by periods of low incidence and the same seasonal months of the successive calendar years. Thus, our analysis was fit for assessing differences between and within- waves, as well as for detecting possible seasonal effects. Our segmentation of time periods did not necessarily coincide with episodes defined by the prevailing SARS-CoV-2 variants. There was a rough association with specific strains (see Fig. 1), but there was also a considerable degree in overlap, especially shortly after new strains were introduced. We were able to adjust the logistic regression analysis on in-hospital mortality for both important patient characteristics and ICU occupancy rate. We analyzed hospital death and treatment duration as separate endpoints, as this nicely corresponds with the clinical aim of discharging the patient alive and with the notion that a longer stay at the ICU or hospital followed by hospital death does not reflect any clinical or survival benefit. Thus, putting both endpoints in the framework of a survival analysis may create an unsolvable contradiction. The absence of an unfavorable effect of a high ICU occupancy rate on the mortality among COVID-19 patients evokes the question whether the high strain on the health care system may have had harmful effects for other patients for whom the treatment (for example elective surgery) was cancelled or postponed. The data made it possible to make a distinction between those patients with an APACHE-IV reason for admission indicative of COVID-19 and those for whom a positive test of SARS-CoV-2 infection was a secondary finding. A limitation of the study is the observational nature, which may imply that unmeasured confounders may have influenced the results. In addition, no data on used medication, and details on the treatment protocol, were present. Dexamethasone and tocilizumab became standard treatment on the ICU in the Netherlands in the early months of 2021 after publication of the pertinent trials [30, 31]. Other treatments such as antiviral medication with remdesivir were less in use. We may conclude that introduction of these new treatment modalities did not prevent the high mortality rate during the third wave. In the beginning of the pandemic, patients in the Netherlands were usually intubated at admission, as the spreading of contagious aerosols by high-flow nasal oxygen was an issue of discussion [32]. After the initial phase, treatment with high-flow nasal oxygen increased as associated risks turned out better than feared.

We used the APACHE-IV for adjustment of severity of illness but the APACHE-IV prediction model was developed long before the COVID-19 pandemic, and does not take infection with SARS-CoV-2 and the different variants properly into account. Thus, the results of our study might suggest the need for regularly updating the APACHE model. The ICU occupancy rate could be calculated with high accuracy for each calendar day and per ICU. However, it was a crude metric when regarding as proxy for (imminent) overload or strain burden, as data on number of patients per nurse, workload per nurse, availability of certificated ICU nurses, absence through illness, the mental status and stress level of the nurses and doctors, and the number of operational ICU beds were not available. The outcomes were the short-term in-hospital mortality and ICU length of stay. Further studies are needed to examine the mortality rates and causes of death of critically ill COVID-19 patients after survival of the hospital treatment trajectory and possible time trends. Especially in the early phases of the pandemic a few cases were diagnosed with a CT scan consistent with COVID-19 without confirmation with a positive PCR test. As there was a high a priori probability of SARS-CoV-2 infection during the first wave, the risk of misclassification was probably limited. Our analysis was restricted to patients in the Netherlands, and thus, our results may not be extrapolated to other countries. An important limitation was the lack of information on vaccination status, which is not registered in the NICE database. We may argue that for those patients who were vaccinated prior to ICU admission the vaccine was not effective (enough), as it obviously did not prevent a clinical course that resulted in admission at the ICU. Thus, the relevance of information on vaccination among ICU patients during the three waves may be limited for a study that aimed to assess mortality risk after ICU admission, in contrast to vaccine efficacy preventing admission. Even in the absence of data on vaccination stratus, we may conclude that despite the increasing vaccination rates from January 2021 onwards, this did not translate in decreasing mortality rates among those COVID-19 patients who were in urgent need of critical care, which was also found by Naouri et al. [24]. Information on vaccination status may be important especially for patients with a comorbid disorder that impairs the vaccine efficacy. The substantially increased prevalence of immune insufficiency during the last endemic episodes highlights this point.

## Conclusions

We found an increase in the mortality rate during the initial upsurge of the third wave after the comparatively low mortality rate during the final upsurge of the second wave. The variation in mortality rates among critically ill COVID-19 patients across the calendar time periods is not explained by differences in case-mix and ICU occupancy rates or by varying proportions of patients with COVID-19 as main reason for ICU admission. The consistent increase in mortality during the initial, rising phase of the three separate waves might be caused by the increased virulence of the contemporary virus strain and lacking immunity to the new strain, next to unmeasured patient-, treatment- and healthcare system characteristics.

### Supplementary Information

**Additional file 1: Figure S1.** Mean occupancy rate at the ICU during the consecutive stages of the COVID-19 pandemic. **Table S1.** Baseline characteristics in group of patients in the COVID-19 registry with MDS record linkage, restricted to those with an APACHE-IV diagnosis indicative of COVID-19 as main reason for ICU admission (*N* = 16,187). Table S2 Treatment characteristics and outcomes, restricted to those with an APACHE-IV diagnosis indicative of COVID-19 as main reason for ICU admission (*N* = 16,187). **Table S3.** Hazard ratios of ICU discharge during the consecutive stages of the COVID-19 pandemic. **Table S4.** All parameter estimates of the multivariable model on hospital mortality in full sample (*N* = 18,772, records with missing data on one of the covariates were excluded, *N* = 576), see Table 3. **Table S5.** All parameter estimates of the multivariable model on hospital mortality in sample restricted to those with APACHE-IV diagnosis indicative of Covid-19 as main reason for ICU admission (*N* = 16,187, records with missing data on one of the covariates data excluded, *N* = 378), see Table 3.

## Data Availability

De-identified aggregated data used for the study will be made available upon reasonable request.

## Abbreviations

ARDS: Acute Respiratory Distress Syndrome
COVID-19: Corona Virus Disease 2019
ICU: Intensive Care Unit
EHR: Electronic Health Record
NICE: The National Intensive Care Evaluation
OR: Odds Ratio
OR_adj_: Adjusted Odds Ratio
SARS-CoV-2: Severe Acute Respiratory Syndrome Corona Virus-2
RT-PCR: Reverse transcription polymerase chain reaction
CO-RADS: COVID-19 Reporting and Data System
BMI: Body Mass Index
APACHE-IV: Acute Physiology and Chronic Health Evaluation IV
