# Supplementary material for: Characteristics and outcome of COVID-19 patients admitted to the ICU: a nationwide cohort study on the comparison between the consecutive stages of the COVID-19 pandemic in the Netherlands, an update
Source: Ann Intensive Care. 2024 Jan 16;14:11. doi: 10.1186/s13613-023-01238-2 (PMC10792150; doi:10.1186/s13613-023-01238-2)
Supplement: Supplementary file 1 — Additional file 1: Figure S1. Mean occupancy rate at the ICU during the consecutive stages of the COVID-19 pandemic. Table S1. Baseline characteristics in group of patients in the COVID-19 registry with MDS record linkage, restricted to those with an APACHE-IV diagnosis indicative of COVID-19 as main reason for ICU admission (N = 16,187). Table S2 Treatment characteristics and outcomes, restricted to those with an APACHE-IV diagnosis indicative of COVID-19 as main reason for ICU admission (N = 16,187). Table S3. Hazard ratios of ICU discharge during the consecutive stages of the COVID-19 pandemic. Table S4. All parameter estimates of the multivariable model on hospital mortality in full sample (N = 18,772, records with missing data on one of the covariates were excluded, N = 576), see Table 3. Table S5. All parameter estimates of the multivariable model on hospital mortality in sample restricted to those with APACHE-IV diagnosis indicative of Covid-19 as main reason for ICU admission (N = 16,187, records with missing data on one of the covariates data excluded, N = 378), see Table 3. [file 13613_2023_1238_MOESM1_ESM.docx]

Supplement Methods

Definition of the various comorbidities (for details see https://www.stichting-nice.nl/)

Immune insufficiency: presence of diagnosis of impairment of humoral or cellular immune function, recent use of immune suppressive therapy, including chemotherapeutics and radiotherapy, presence of aids diagnosis (that is, hiv positive in combination with clinical diagnosis and/ or CD4 count <200).

Renal insufficiency: increased creatinine >177umol/L, diagnosis of chronic renal insufficiency and/ or chronic treatment with peritoneal or hemodialysis in medical history

Respiratory insufficiency: various restrictive, obstructive, and vascular lung disorders leading to severe limitations in physical activity, chronic hypoxia, secondary polycythemia, severe pulmonary hypertension, O2-dependent respiratory disorders, sarcoidosis.

Cardiovascular insufficiency: symptoms of angina pectoris during rest or minimal physical strain, NYHA IV

Malignancy: oncology with presence of distant metastasis, stage IV cancer, hematological malignancies

Liver cirrhosis: diagnosis based on pathology report and presence of portal hypertension, hepatic insufficiency or encephalopathy

Supplement Figure 1 Mean occupancy rate at the ICU during the consecutive stages of the COVID-19 pandemic


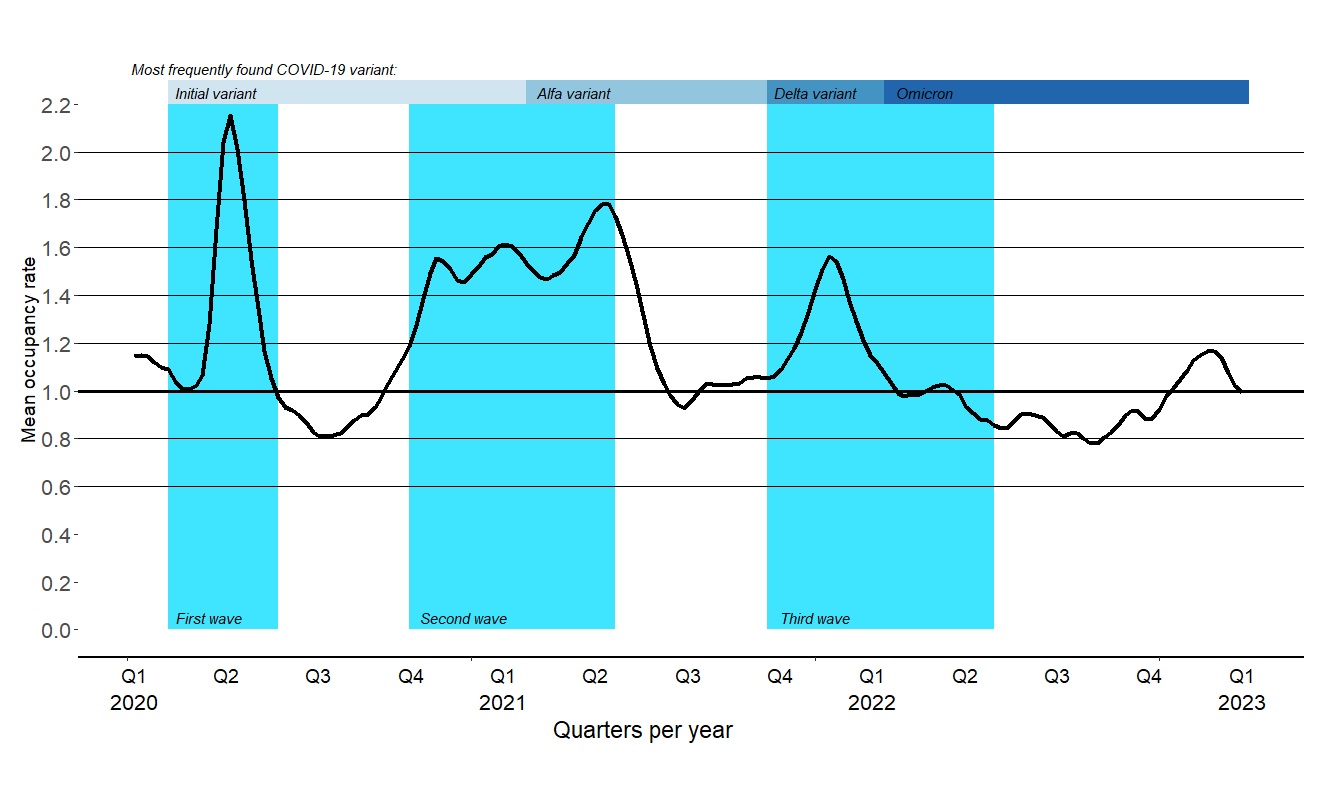


Supplement Table 1 Baseline characteristics in group of patients in the COVID-19 registry with MDS record linkage, restricted to those with an APACHE-IV diagnosis indicative of COVID-19 as main reason for ICU admission (N=16,187)

|  | 1.Wave 1  01/02/2020-  24/05/2020 | 2.In-between  25/05/2020-04/10/2020 | 3.Wave 2.1  05/10/2020-  31/01/2021 | 4.Wave 2.2  01/02/2021-  24/05/2021 | 5.In-between  25/05/2021-  04/10/2021 | 6.Wave 3.1  05/10/2021-  31/01/2022 | 7.Wave 3.2  01/02/2022-  24/05/2022 | 8.In-between  25/05/2022-  04/10/2022 | 9.Endemic  05/10/2022-  31/01/2023 |
| --- | --- | --- | --- | --- | --- | --- | --- | --- | --- |
| Number of patients  with MDS linkage: N | 2,550 | 424 | 3,940 | 4,337 | 1,179 | 2,710 | 589 | 207 | 251 |
|  |  |  |  |  |  |  |  |  |  |
| Mean age (years) (SD) | 63.1 (11.0) | 61.9 (12.6) | 64.3 (10.9) | 61.4 (11.4) | 55.6 (14.0) | 60.4 (12.7) | 64.2 (12.9) | 65.5 (14.2) | 66.3 (13.8) |
|  |  |  |  |  |  |  |  |  |  |
| Gender: Male N (%) | 1846 (72.4) | 283 (66.7) | 2845 (72.2) | 2922 (67.4) | 720 (61.1) | 1813 (66.9) | 353 (59.9) | 132 (63.8) | 165 (65.7) |
| Comorbidity: N (%): |  |  |  |  |  |  |  |  |  |
| Immune insufficiency | 193 (7.6) | 29 (6.8) | 417 (10.6) | 384 (8.9) | 119 (10.1) | 294 (10.8) | 181 (30.7) | 68 (32.9) | 70 (27.9) |
| Renal insufficiency | 64 (2.5) | 14 (3.3) | 230 (5.8) | 144 (3.3) | 40 (3.4) | 114 (4.2) | 62 (10.5) | 24 (11.6) | 37 (14.7) |
| Respiratory insufficiency | 285 (11.2) | 44 (10.4) | 581 (14.7) | 533 (12.3) | 115 (9.8) | 297 (11) | 139 (23.6) | 58 (28.0) | 87 (34.7) |
| Cardiovascular | 27 (1.1) | 6 (1.4) | 66 (1.7) | 55 (1.3) | 20 (1.7) | 28 (1.0) | 23 (3.9) | 8 (3.9) | 13 (5.2) |
| Malignancy | 57 (2.2) | 10 (2.4) | 130 (3.3) | 72 (1.7) | 29 (2.5) | 68 (2.5) | 62 (10.5) | 26 (12.6) | 32 (12.7) |
| Liver cirrhosis | 3 (0.1) | 2 (0.5) | 18 (0.5) | 20 (0.5) | 4 (0.3) | 14 (0.5) | 4 (0.7) | 5 (2.4) | 4 (1.6) |
| Diabetes Mellitus | 476 (18.7) | 134 (31.6) | 1032 (26.2) | 928 (21.4) | 252 (21.4) | 572 (21.1) | 130 (22.1) | 41 (19.8) | 52 (20.7) |
| At least 1 of these N (%) | 896 (35.1) | 191 (45.0) | 1841 (46.7) | 1690 (39.0) | 443 (37.6) | 1022 (37.7) | 378 (64.2) | 144 (69.6) | 180 (71.7) |
|  |  |  |  |  |  |  |  |  |  |
| Mean BMI (kg/m^2^) (SD) | 28.77 (5.01) | 29.73 (5.6) | 29.57 (5.35) | 30.18 (5.78) | 30.57 (6.4) | 29.93 (5.73) | 27.74 (6.34) | 26.8 (6.2) | 26.97 (6.75) |
| BMI >30 N (%) | 792 (31.1) | 168 (39.6) | 1562 (39.6) | 1899 (43.8) | 536 (45.5) | 1128 (41.6) | 165 (28) | 44 (21.3) | 54 (21.5) |
|  |  |  |  |  |  |  |  |  |  |
| Mean APACHE-III (SD) | 59.5 (20.63) | 59.55 (20.57) | 63.06 (19.7) | 59.44 (18.12) | 56.9 (18.36) | 60.49 (19.92) | 66.86 (22.54) | 71.18 (26.45) | 68.96 (23.77) |
|  |  |  |  |  |  |  |  |  |  |
| 1.ARDS (APACHE-IV) N(%) | 429 (16.8) | 56 (13.2) | 437 (11.1) | 609 (14) | 156 (13.2) | 420 (15.5) | 55 (9.3) | 5 (2.4) | 11 (4.4) |
| 2.PaO2/FiO2 ratio <300 | 2238 (87.8) | 361 (85.1) | 3595 (91.2) | 4026 (92.8) | 1077 (91.3) | 2477 (91.4) | 484 (82.2) | 156 (75.4) | 194 (77.3) |
| 1. and/ or 2. N (%) | 2298 (90.1) | 370 (87.3) | 3609 (91.6) | 4047 (93.3) | 1082 (91.8) | 2487 (91.8) | 484 (82.2) | 156 (75.4) | 194 (77.3) |

Supplement Table 2 Treatment characteristics and outcomes, restricted to those with an APACHE-IV diagnosis indicative of COVID-19 as main reason for ICU admission (N=16,187)

|  | 1.Wave 1  01/02/2020-  24/05/2020 | 2.In-between  25/05/2020-04/10/2020 | 3.Wave 2.1  05/10/2020-  31/01/2021 | 4.Wave 2.2  01/02/2021-  24/05/2021 | 5.In-between  25/05/2021-  04/10/2021 | 6.Wave 3.1  05/10/2021-  31/01/2022 | 7.Wave 3.2  01/02/2022-  24/05/2022 | 8.In-between  25/05/2022-  04/10/2022 | 9.Endemic  05/10/2022-  31/01/2023 |
| --- | --- | --- | --- | --- | --- | --- | --- | --- | --- |
| N MDS with record linkage | 2,550 | 424 | 3,940 | 4,337 | 1,179 | 2,710 | 589 | 207 | 251 |
| Mean PaO2 (mmHg) t=0 (SD) | 84.9 (36.26) | 75.86 (24.1) | 75.53 (28.28) | 75.84 (29.59) | 75.4 (28.32) | 75.56 (30.54) | 78.58 (34.83) | 80.89 (39.45) | 80.84 (34.57) |
|  |  |  |  |  |  |  |  |  |  |
| Mech. ventil. t=0 (N,%) | 1,229 (48.2) | 83 (19.6) | 934 (23.7) | 1,011 (23.3) | 237 (20.1) | 676 (24.9) | 138 (23.4) | 66 (31.9) | 88 (35.1) |
| Mech. ventil. t=24h (N,%) | 2,054 (80.5) | 227 (53.5) | 2,333 (59.2) | 2,572 (59.3) | 642 (54.5) | 1,604 (59.2) | 289 (49.1) | 94 (45.4) | 131 (52.2) |
| Vasoactive drugs (N,%) | 1,736 (68.1) | 178 (42.0) | 1,900 (48.2) | 2,020 (46.6) | 481 (40.8) | 1,286 (47.5) | 229 (38.9) | 75 (36.2) | 95 (37.8) |
|  |  |  |  |  |  |  |  |  |  |
| Acute renal failure (N,%) | 221 (8.7) | 22 (5.2) | 243 (6.2) | 235 (5.4) | 41 (3.5) | 156 (5.8) | 61 (10.4) | 26 (12.6) | 29 (11.6) |
|  |  |  |  |  |  |  |  |  |  |
| Mean bed occupancy (SD) | 1.83 (0.62) | 1.09 (0.4) | 1.51 (0.45) | 1.65 (0.44) | 1.08 (0.32) | 1.36 (0.38) | 1.03 (0.36) | 0.9 (0.3) | 1.06 (0.45) |
|  |  |  |  |  |  |  |  |  |  |
| Transfer to other hospital (N,%) | 851 (33.4) | 107 (25.2) | 1166 (29.6) | 1267 (29.2) | 309 (26.2) | 771 (28.5) | 82 (13.9) | 19 (9.2) | 19 (7.6) |
|  |  |  |  |  |  |  |  |  |  |
| Mean length of pre ICU hospital stay in days (SD) | 1.7 (2.7) | 1.9 (4.6) | 2.3 (8.8) | 2.1 (2.9) | 2.1 (3.3) | 2.2 (8.1) | 4.4 (32.8) | 1.8 (3.7) | 4.9 (49.2) |
|  |  |  |  |  |  |  |  |  |  |
| Mean length of stay ICU (SD) | 21.25 (20.63) | 17.17 (15.99) | 18.12 (17.92) | 16.93 (16.98) | 16.23 (25.47) | 16.49 (21.92) | 12.65 (23.76) | 9.01 (11.46) | 8.68 (14.44) |
| Median length of stay ICU  (IQR) | 16  (9-29) | 13  (6-22) | 12  (7-24) | 11  (6-22) | 10  (6-20.5) | 11  (6-22) | 7  (3-15) | 5  (3-10) | 5  (3-10) |
|  |  |  |  |  |  |  |  |  |  |
| Mean length of stay in days hospital (SD) *^1^ | 40.2 (121.8) | 42.8 (140.0) | 36.8 (108.5) | 36.1 (101.8) | 26.3 (56.5) | 28.3 (60.3) | 22.1 (42.5) | 19.5 (35.5) | 18.7 (32.0) |
| Hospital death (N, %) | 748 (29.3) | 112 (26.4) | 1,255 (31.9) | 1,017 (23.4) | 234 (19.8) | 762 (28.1) | 188 (31.9) | 54 (26.1) | 66 (26.3) |

*^1^ calculated since data of admission at the ICU

Supplement Table 3 Hazard ratios of ICU discharge during the consecutive stages of the COVID-19 pandemic

|  | 1.Wave 1  01/02/2020-  24/05/2020  Reference | 2.In-between  25/05/2020-04/10/2020 | 3.Wave 2.1  05/10/2020-  31/01/2021 | 4.Wave 2.2  01/02/2021-  24/05/2021 | 5.In-between  25/05/2021-  04/10/2021 | 6.Wave 3.1  05/10/2021-  31/01/2022 | 7.Wave 3.2  01/02/2022-  24/05/2022 | 8.In-between  25/05/2022-  04/10/2022 | 9.Endemic  05/10/2022-  31/01/2023 |
| --- | --- | --- | --- | --- | --- | --- | --- | --- | --- |
|  |  |  |  |  |  |  |  |  |  |
| Total (N=18,772) |  |  |  |  |  |  |  |  |  |
| 1. Crude | 1.00 | 1.34 | 1.16 | 1.35 | 1.51 | 1.38 | 2.37 | 3.07 | 3.17 |
|  | [1.00 – 1.00] | [1.2 – 1.5] | [1.1-1.23] | [1.28 – 1.43] | [1.4 – 1.63] | [1.29 – 1.46] | [2.19-2.57] | [2.77-3.42] | [2.86 – 3.52] |
| Wald X2, df, P value |  |  |  |  |  |  |  | 1094.65, 8, | <0.001 |
| 2. Adjusted for age, sex | 1.00 | 1.35 | 1.3 | 1.43 | 1.39 | 1.37 | 2.59 | 3.01 | 3.35 |
| BMI, and APACHE-IV risk | [1.00 – 1.00] | [1.2-1.51] | [1.23-1.38] | [1.35-1.51] | [1.29-1.5] | [1.29-1.46] | [2.38-2.81] | [2.7-3.35] | [3.01-3.72] |
| Wald X2, df, P value |  |  |  |  |  |  |  | 1023.55, 8, | <0.001 |
| 3. Adjusted for (…), and | 1.00 | 1.27 | 1.27 | 1.41 | 1.31 | 1.32 | 2.43 | 2.81 | 3.14 |
| ICU occupancy rate | [1.00 – 1.00] | [1.13-1.43] | [1.2-1.35] | [1.33-1.49] | [1.2-1.42] | [1.24-1.41] | [2.23-2.66] | [2.51-3.15] | [2.82-3.51] |
| Wald X2, df, P value |  |  |  |  |  |  |  | 808.196, 8, | <0.001 |
|  |  |  |  |  |  |  |  |  |  |
| Restricted to those with APACHE-IV diagnosis indicative of Covid-19 as main reason for ICU admission (N=16,187) | | | | | | | |  |  |
| 1. Crude | 1.00 | 1.29 | 1.14 | 1.35 | 1.54 | 1.34 | 1.75 | 2.81 | 2.85 |
|  | [1.00 – 1.00] | [1.14-1.45] | [1.07-1.21] | [1.27-1.43] | [1.43-1.67] | [1.25-1.42] | [1.58-1.95] | [2.39-3.31] | [2.45-3.31] |
| Wald X2, df, P value |  |  |  |  |  |  |  | 449.924, 8, | <0.001 |
| 2. Adjusted for age, sex | 1.00 | 1.3 | 1.26 | 1.41 | 1.39 | 1.32 | 2.03 | 3.13 | 3.25 |
| BMI, and APACHE-IV risk | [1.00 – 1.00] | [1.15-1.46] | [1.19-1.34] | [1.33-1.49] | [1.28-1.5] | [1.24-1.41] | [1.82-2.25] | [2.65-3.69] | [2.79-3.78] |
| Wald X2, df, P value |  |  |  |  |  |  |  | 478.886, 8, | <0.001 |
| 3. Adjusted for (…), and | 1.00 | 1.24 | 1.24 | 1.39 | 1.33 | 1.28 | 1.94 | 2.99 | 3.1 |
| ICU occupancy rate | [1.00 – 1.00] | [1.1-1.4] | [1.16-1.31] | [1.31-1.48] | [1.22-1.44] | [1.2-1.37] | [1.74-2.17] | [2.52-3.55] | [2.66-3.63] |
| Wald X2, df, P value |  |  |  |  |  |  |  | 410.892, 8, | <0.001 |
|  |  |  |  |  |  |  |  |  |  |
|  |  |  |  |  |  |  |  |  |  |

Supplement Table 4, all parameter estimates of the multivariable model on hospital mortality In full sample (N=18,772, records with missing data on one of the covariates were excluded, N=576), see Table 3

|  |  | 95%-Confidence Interval | Chi-square, df | P Value |
| --- | --- | --- | --- | --- |
| Episode |  |  |  |  |
| 1. Wave 1 | 1.00 (ref) |  | 82.0, 8 | <0.0001 |
| 2. In-between | 0.86 | 0.67 – 1.11 |  |  |
| 3. Wave 2.1 | 0.98 | 0.87 – 1.10 |  |  |
| 4. Wave 2.2 | 0.78 | 0.69 – 0.88 |  |  |
| 5. In-between | 0.79 | 0.65 – 0.95 |  |  |
| 6. Wave 3.1 | 1.01 | 0.88 – 1.16 |  |  |
| 7. Wave 3.2 | 0.70 | 0.58 – 0.84 |  |  |
| 8. In-between | 0.50 | 0.39 – 0.65 |  |  |
| 9. Endemic | 0.52 | 0.41 – 0.66 |  |  |
| Age (missing: N=1) |  |  |  |  |
| 1. <40 years | 1.00 (ref) |  | 646.0, 10 | <0.0001 |
| 2. 40-45 | 1.59 | 1.06 – 2.37 |  |  |
| 3. 45-50 | 1.40 | 0.98 – 2.00 |  |  |
| 4. 50- 55 | 2.03 | 1.48 – 2.77 |  |  |
| 5. 55-60 | 2.48 | 1.84 – 3.35 |  |  |
| 6. 60-65 | 3.47 | 2.59 – 4.65 |  |  |
| 7. 65-70 | 4.84 | 3.62 – 6.47 |  |  |
| 8. 70-75 | 5.98 | 4.47 – 7.99 |  |  |
| 9. 75-80 | 7.40 | 5.50 – 9.95 |  |  |
| 10. 80-85 | 9.51 | 6.82 – 13.26 |  |  |
| 11. >85 | 11.08 | 6.74 – 18.22 |  |  |
| Gender |  |  |  |  |
| 1. Male | 1.00 (ref) |  | 25.5, 1 | <0.0001 |
| 2. Female | 0.81 | 0.75 – 0.88 |  |  |
| BMI (missing: N=466) |  |  |  |  |
| 1. <18.5 kg/m^2^ | 1.00 (ref) |  | 18.7, 5 | 0.0022 |
| 2. 18.5 – 25 | 0.64 | 0.44-0.93 |  |  |
| 3. 25 – 30 | 0.60 | 0.42-0.88 |  |  |
| 4. 30 – 35 | 0.58 | 0.39-0.84 |  |  |
| 5. 35 – 40 | 0.63 | 0.43-0.93 |  |  |
| 6. >40 | 0.79 | 0.53-1.19 |  |  |
| APACHE-IV prob (quintiles) (missing: N=84) | | |  |  |
| 1. 0 - <11.5% | 1.00 (ref) |  | 1121.1, 4 | <0.0001 |
| 2. 11.5 - <18.4% | 1.69 | 1.45-1.97 |  |  |
| 3. 18.4 - <26.3% | 2.39 | 2.06-2.77 |  |  |
| 4. 26.3-<39.0% | 3.40 | 2.94-3.93 |  |  |
| 5. 39.0-<=98.89% | 7.71 | 6.67-8.90 |  |  |
| Occupancy rate (quintiles) (missing: N=89) | | |  |  |
| 1. 0.064 - <=1.017 | 1.00 (ref) |  | 4.3, 4 | 0.38 |
| 2. 1.017- <=1.265 | 0.95 | 0.84-1.07 |  |  |
| 3. 1.265- <=1.512 | 0.90 | 0.79-1.02 |  |  |
| 4. 1.512- <=1.867 | 0.92 | 0.80-1.04 |  |  |
| 5. 1.867-<=5.486 | 0.88 | 0.77-1.01 |  |  |
|  |  |  |  |  |

Supplement Table 5, all parameter estimates of the multivariable model on hospital mortality in sample restricted to those with APACHE-IV diagnosis indicative of Covid-19 as main reason for ICU admission (N=16,187, records with missing data on one of the covariates data excluded, N=378), see Table 3

|  |  | 95%-Confidence Interval | Chi-square, df | P Value |
| --- | --- | --- | --- | --- |
| Episode |  |  |  |  |
| 1. Wave 1 | 1.00 (ref) |  | 54.6, 8 | <0.0001 |
| 2. In-between | 0.85 | 0.65-1.11 |  |  |
| 3. Wave 2.1 | 0.98 | 0.87-1.11 |  |  |
| 4. Wave 2.2 | 0.80 | 0.71-0.91 |  |  |
| 5. In-between | 0.81 | 0.67-0.99 |  |  |
| 6. Wave 3.1 | 1.03 | 0.89-1.18 |  |  |
| 7. Wave 3.2 | 0.81 | 0.64-1.01 |  |  |
| 8. In-between | 0.48 | 0.33-0.69 |  |  |
| 9. Endemic | 0.48 | 0.34-0.68 |  |  |
| Age (missing: N=1) |  |  |  |  |
| 1. <40 years | 1.00 (ref) |  | 674.6, 10 | <0.0001 |
| 2. 40-45 | 1.95 | 1.21-3.14 |  |  |
| 3. 45-50 | 1.72 | 1.12-2.64 |  |  |
| 4. 50- 55 | 2.53 | 1.72-3.72 |  |  |
| 5. 55-60 | 3.22 | 2.22-4.66 |  |  |
| 6. 60-65 | 4.67 | 3.24-6.72 |  |  |
| 7. 65-70 | 6.76 | 4.71-9.71 |  |  |
| 8. 70-75 | 8.86 | 6.16-12.74 |  |  |
| 9. 75-80 | 11.15 | 7.71-16.13 |  |  |
| 10. 80-85 | 16.52 | 10.94-24.96 |  |  |
| 11. >85 | 25.79 | 12.13-54.82 |  |  |
| Gender |  |  |  |  |
| 1. Male | 1.00 (ref) |  | 29.1, 1 | <0.0001 |
| 2. Female | 0.79 | 0.72-0.86 |  |  |
| BMI (missing: N=300) |  |  | 16.3, 5 | 0.0059 |
| 1. <18.5 kg/m^2^ | 1.00 (ref) |  |  |  |
| 2. 18.5 – 25 | 0.72 | 0.45-1.16 |  |  |
| 3. 25 – 30 | 0.68 | 0.42-1.08 |  |  |
| 4. 30 – 35 | 0.68 | 0.42-1.09 |  |  |
| 5. 35 – 40 | 0.75 | 0.46-1.22 |  |  |
| 6. >40 | 0.96 | 0.58-1.58 |  |  |
| APACHE-IV prob (quintiles) |  |  |  |  |
| 1. 0 - <11.5% | 1.00 (ref) |  | 635.7, 4 | <0.0001 |
| 2. 11.5 - <18.4% | 1.51 | 1.27-1.78 |  |  |
| 3. 18.4 - <26.3% | 2.01 | 1.71-2.36 |  |  |
| 4. 26.3-<39.0% | 2.68 | 2.28-3.16 |  |  |
| 5. 39.0-<=98.89% | 5.51 | 4.68-6.49 |  |  |
| Occupancy rate (quintiles) (missing: N=81) | | |  |  |
| 1. 0.064 - <=1.017 | 1.00 (ref) |  | 3.8, 4 | 0.43 |
| 2. 1.017- <=1.265 | 0.93 | 0.81-1.07 |  |  |
| 3. 1.265- <=1.512 | 0.89 | 0.77-1.02 |  |  |
| 4. 1.512- <=1.867 | 0.91 | 0.79-1.05 |  |  |
| 5. 1.867-<=5.486 | 0.88 | 0.76-1.01 |  |  |
|  |  |  |  |  |
